# Rosai-Dorfman Disease in a Rare Cardiac Presentation: Left Ventricular Mass Causing Ventricular Tachycardia

**DOI:** 10.1016/j.jaccas.2026.107608

**Published:** 2026-03-25

**Authors:** Motaz Al Yafi, Swetha Pasala, Valentina Robila, Cory Trankle, Bharadhwaj Kolipakkam, Patricia Nicolato

**Affiliations:** a Division of Cardiothoracic Surgery, Department of Surgery, Pauley Heart Center, Virginia Commonwealth University, Richmond, Virginia, USA; b Division of Cardiology, Department of Internal Medicine, Pauley Heart Center, Virginia Commonwealth University, Richmond, Virginia, USA; c Department of Pathology, Virginia Commonwealth University, Richmond, Virginia, USA; d Division of Hematology/Oncology, Department of Internal Medicine, Virginia Commonwealth University, Richmond, Virginia, USA

**Keywords:** cardiac magnetic resonance, left ventricle, palpitations, shortness of breath, treatment, ventricular tachycardia

## Abstract

**Background:**

Rosai-Dorfman disease (RDD) is a rare, benign histiocytic disorder that most often presents with nodal involvement. Cardiac involvement is exceedingly uncommon and poses significant diagnostic and therapeutic challenges.

**Case Summary:**

A 51-year-old woman presented with shortness of breath and palpitations and was found to have monomorphic ventricular tachycardia. Imaging revealed a left ventricular mass, and biopsy confirmed RDD. The lesion was surgically excised with cryoablation and CorMatrix reinforcement. The patient recovered uneventfully and remains disease free 25 months postoperatively.

**Discussion:**

RDD with cardiac involvement has been reported in only approximately 25 cases, with very few affecting the left ventricle. This case demonstrates how comprehensive imaging and deep myocardial biopsy are essential for accurate diagnosis. Surgical resection remains the treatment of choice for localized disease.

**Take-Home Messages:**

Cardiac RDD, though rare, should be included in the differential diagnosis of infiltrative cardiac masses. Complete excision offers curative potential.

## Visual Summary

**Figure:**
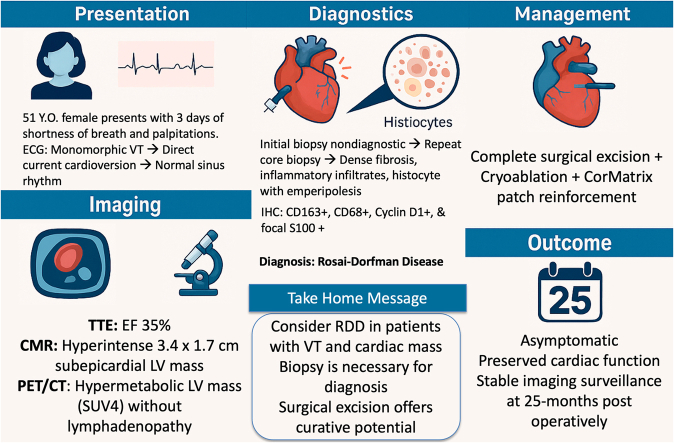

## History of Presentation

A 51-year-old woman presented to the emergency department with 3 days of shortness of breath and palpitations. She was found to be in monomorphic ventricular tachycardia (VT) with a heart rate of 180 beats/min. She underwent direct current cardioversion and was restored to normal sinus rhythm. Cardiac magnetic resonance (CMR) showed a left ventricular (LV) mass measuring 3.4 × 1.7 cm with early and delayed contrast enhancement.

**Take-Home Messages**

• This case highlights the importance of considering RDD in the differential diagnosis of patients presenting with unexplained ventricular tachycardia and cardiac masses.
• Deep myocardial biopsy to achieve a definitive diagnosis may be necessary.
• A multidisciplinary approach to diagnosis and management is crucial.
• Complete surgical excision can result in resolution of arrhythmia and excellent long-term outcomes in RDD with localized cardiac involvement.

## Past Medical History

She had no significant past medical history and did not take any medications before this presentation. She had no prior episodes of VT.

## Differential Diagnosis

The differential diagnosis encompasses a wide range of possibilities, including primary sarcoma, lymphoma, and benign infiltrative tumors such as fibroma, lipoma, and hemangioma. Less commonly, it may be attributed to inflammatory masses associated with conditions such as cardiac sarcoidosis or giant cell myocarditis.

## Investigations

**Figure 1:**
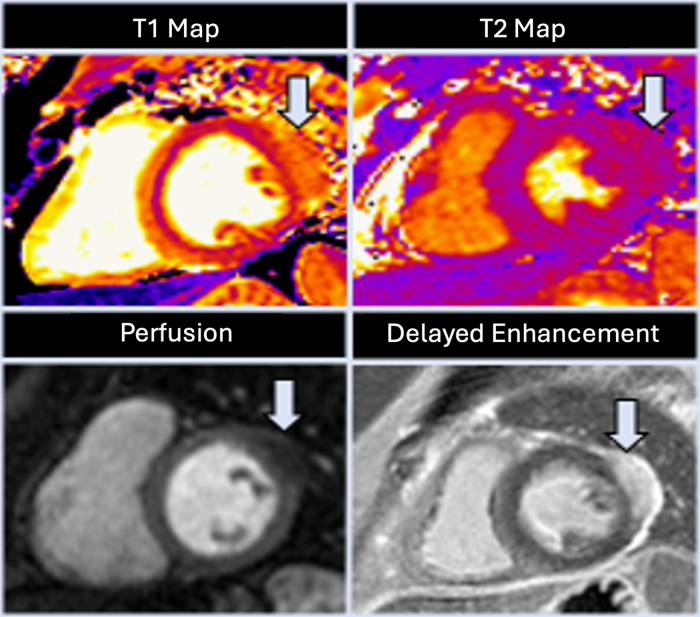
Preoperative Cardiac Magnetic Resonance Cardiac magnetic resonance demonstrating a subepicardial mass along the lateral wall, with elevated native T1 (top left) and T2 (top right) values. There was heterogeneous contrast uptake on first-pass perfusion images (bottom left), with significant enhancement on delayed postcontrast imaging (bottom right).

**Figure 2:**
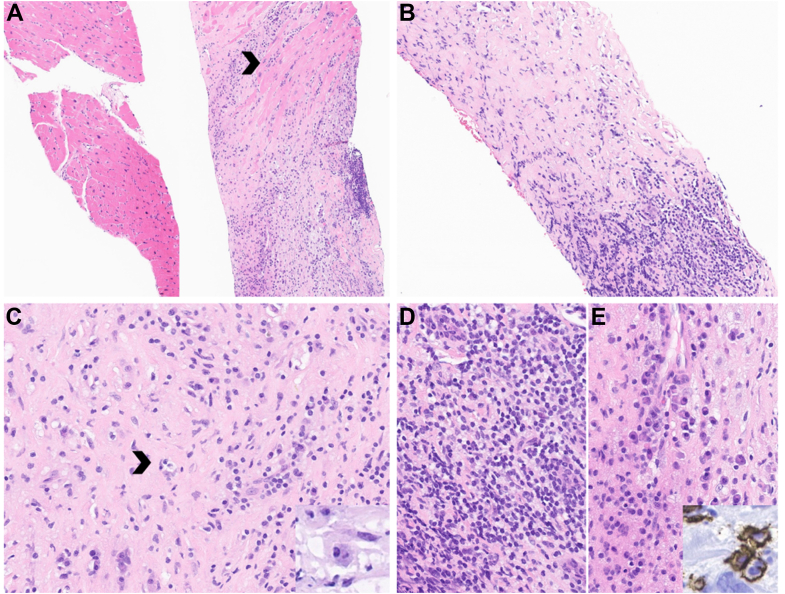
Cardiac Mass Biopsy Results (A) Cardiac biopsies showing patchy involvement, with an inflammatory process (right core) infiltrating residual cardiac myocytes (arrowhead) and only small areas of uninvolved myocardium (left core) (4×). (B) The mass-like lesion consists of dense fibrosis, with aggregates and scattered inflammatory cells (10×). (C) The histiocytes have enlarged nuclei, foamy cytoplasm, and a subset show emperipolesis (arrowhead and inset) (20×). Dense inflammatory infiltrates consist of lymphocytes (D) admixed with polyclonal plasma cells (E), positive for CD138 (inset) (40×). The histiocytes are positive for macrophage lineage markers CD163, Oct2, and CD68 (not shown). BRAF V600E was negative (not shown).

Autoimmune and malignant disorders were screened with detailed history, physical examination, and laboratory evaluation, which were unrevealing. A transthoracic echocardiogram revealed an exophytic mass on the lateral wall of the LV with reduced systolic function (ejection fraction 35%). CMR confirmed a 3.4 × 1.7 cm subepicardial-based mass along the lateral wall. The mass appeared inseparable from the adjacent myocardium and appeared slightly hyperintense on T2-weighted imaging. Further images showed an early heterogeneous uptake of contrast on first pass perfusion images and diffuse enhancement on delayed postcontrast images (Figure 1). Positron emission tomography/computed tomography showed increased hypermetabolic activity along the midsegment of the anterolateral wall of the LV measuring a standardized uptake value of 4 without any lymphadenopathy or other extranodal foci of disease. A bone marrow biopsy was negative for lymphoma or leukemia. In preparation for intervention, the patient underwent a left heart catheterization, which revealed no significant coronary artery disease. The decision was then made to proceed with biopsy of the LV mass. An initial attempt at biopsy was made through a left anterior mini-thoracotomy with a punch biopsy that traversed the anterior layer of the tumor, and pathology results showed fibrovascular tissue with lymphoplasmacytic infiltrate. After a period of recovery, we proceeded with a repeated attempt to biopsy the LV mass this time through a left lateral mini-thoracotomy. This allowed us to obtain adequate core biopsies of the LV mass in the myocardium using a true cut biopsy instrument in a perpendicular fashion to the mass. Multiple core biopsies demonstrated the mass-like process, its infiltrative margins, and only small focal areas on uninvolved myocardium (Figure 2A). The mass consisted of dense fibrosis with prominent inflammatory infiltrates as well as scattered histiocytes. The inflammatory aggregates were composed of a mixture of cytologically unremarkable CD20-positive B lymphocytes and CD3-positive T lymphocytes as well as increased CD138-positive plasma cells (Figures 2D and 2E), with a polyclonal pattern of kappa and lambda light chain expression. Altogether, these data excluded the possibility of a lymphoproliferative process. Instead, a prominent population of histiocytes was noted, with enlarged nuclei, dispersed chromatin, small central nucleoli, and abundant foamy cytoplasm. A subset of these histiocytes contained engulfed intact inflammatory cells, known as emperipolesis (Figure 2C). The lesional histiocytes were positive for monocyte and macrophage markers CD163, CD68, and Oct2. In addition, they were positive for cyclin D1, focally positive for S100, while negative for CD1a and factor XIIIa. The immunohistochemical stain BRAF V600E was negative, further arguing against other histiocytic diseases, such as Langerhans cell histiocytosis and Erdheim-Chester disease. Molecular analysis detected no clinically significant variants, and generation sequencing showed no specific mutations such as NRAS, KRAS, MAP2K1, or ARAF. Overall, the histomorphologic features and immunoprofile were consistent with Rosai-Dorfman disease (RDD).

## Management

**Figure 3:**
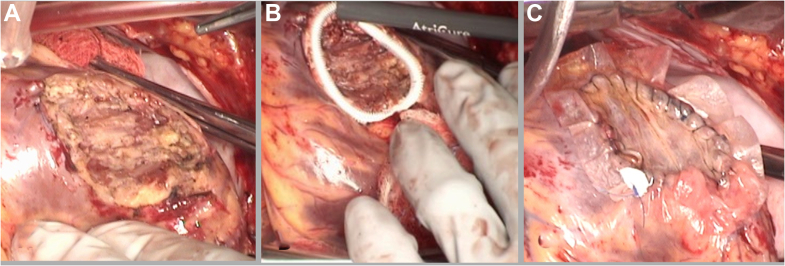
Surgical Management (A) Tumor bed after complete excision with electrocautery. (B) Cryoablation of the tumor margins and tumor bed with the cryoICE cryoprobe. (C) Double-layer CorMatrix extracellular matrix patch reinforcement.

Because of the focal nature of the disease, surgical excision was recommended, and systemic therapy was deferred. The patient's initial symptoms of palpitations were controlled with a β-blocker, and she was otherwise asymptomatic after cardioversion. Work-up for surgical resection with a backup plan for a ventricular support device and transplantation was performed. Once the patient was screened for transplantation, we proceeded with surgical resection. The patient was taken to the operating room, and a median sternotomy was performed. She was placed on cardiopulmonary bypass with bicaval cannulation. The heart was arrested using cardioplegia, and aortic cross-clamp was applied. The mass was noted on the lateral posterior wall of the LV measuring 4 × 3 cm. This was rotated into the field, and the tumor was resected in layers using electrocautery until the tumor was able to be separated from the myocardium (Figure 3A). Once the tumor was completely excised, a thin layer of myocardium remained intact. The edges and base of the tumor bed were treated with cryoablation using the cryoICE cryoprobe (AtriCure) (Figure 3B). The tumor bed was then reinforced with a double layer of CorMatrix Cor PATCH extracellular matrix (CorMatrix Cardiovascular Inc) (Figure 3C). The patient was then weaned from cardiopulmonary bypass and had an uneventful recovery.

## Outcome and Follow-Up

**Figure 4:**
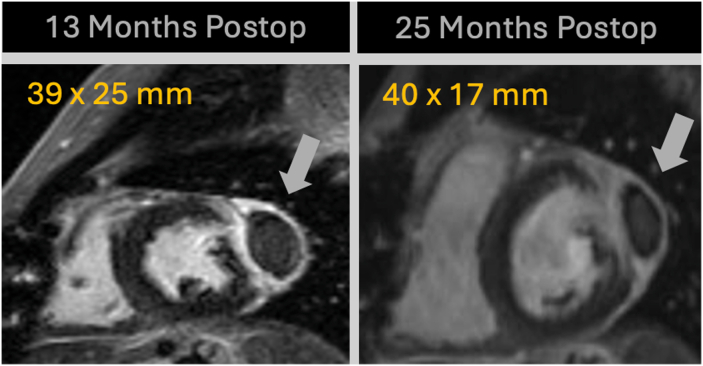
Follow-Up Cardiac Magnetic Resonance at 13 and 25 Months Postoperatively

The patient was discharged home on postoperative day 9 without complications. She has been followed up in clinic at 3, 8, 13, and 25 months postoperatively with serial CMR. Postoperative CMRs persistently demonstrated fluid collection along the epicardial surface of the tumor resection site with inner material that has high T2 signal and the absence of contrast penetration, consistent with CorMatrix material used during surgery (Figure 4).

## Discussion

RDD is a rare histiocytic tumor that most commonly presents in the lymph nodes with extremely rare extranodal presentation occurring in the heart.^1^ There are approximately 25 cardiac cases reported in the literature at the time of this review.^2^, ^3^, ^4^ The right atrium is the most affected site, followed by left atrium, interatrial septum, and only 3 reported cases to involve the LV.^5^^,^^6^^,^^8^ The pathogenesis of RDD is poorly understood. Immunodeficiency states and a variety of infections (human herpesvirus 6, *Cytomegalovirus*, Epstein-Barr virus, *Klebsiella*, *Brucella*) have been suggested as likely etiologies of RDD.^1^ Five cases were found postmortem, and one died during the ultrasound-guided biopsy of the tumor involving the pulmonary artery.^4^^,^^5^ This presents a diagnostic challenge as tissue biopsy must confirm the presence of histiocytic proliferation with emperipolesis and inflammatory cells that are specific for RDD. As in this case, punch biopsies were found to be diagnostic when traversing the tumor into the myocardium. Original biopsies that were tangential to the tumor were nondiagnostic. The clinical presentation of cardiac RDD varies based on the location and extent of involvement. Most reported cases present with chest pain and/or shortness of breath, whereas a smaller number of patients report palpitations and are subsequently found to have an arrhythmia. The development of arrhythmias in cardiac RDD is thought to stem from tissue injury and scarring caused by histiocyte proliferation within the myocardium. This disruption of normal myocardial architecture can interfere with electrical conduction pathways, creating the potential for arrhythmogenic activity.^9^ For patients with resectable lesions, surgical excision is the treatment of choice and results in complete remission; however, extensive involvement of vital organs may cause life-threatening complications.^2^^,^^5^^,^^7^ Among the reported cases with left ventricular involvement, one patient underwent subtotal resection with no evidence of disease progression at 5-month follow-up, and another underwent complete excision of the left ventricular mass but was lost to follow-up after 3 months.^4^ Targeted therapies affecting the rat sarcoma/rapidly accelerated fibrosarcoma/mitogen-activated protein kinase/extracellular signal-regulated kinase pathway have recently been identified as effective therapies particularly for patients with positive mutations.^1^ Therefore, a multidisciplinary approach to diagnosis and management of RDD with cardiac involvement is crucial for the best long-term outcome.

## Conclusions

We present a rare case of RDD involving the LV, manifesting as VT and an infiltrative cardiac mass. Through comprehensive imaging and sequential biopsy, a definitive diagnosis was achieved, emphasizing the importance of deep, targeted sampling in such cases. Successful surgical resection led to complete removal of the lesion and an excellent postoperative recovery. This case underscores the critical role of multidisciplinary collaboration in diagnosing and managing rare cardiac histiocytic disorders, as well as the need for continued surveillance to monitor for recurrence or residual disease.

**Visual Summary:**
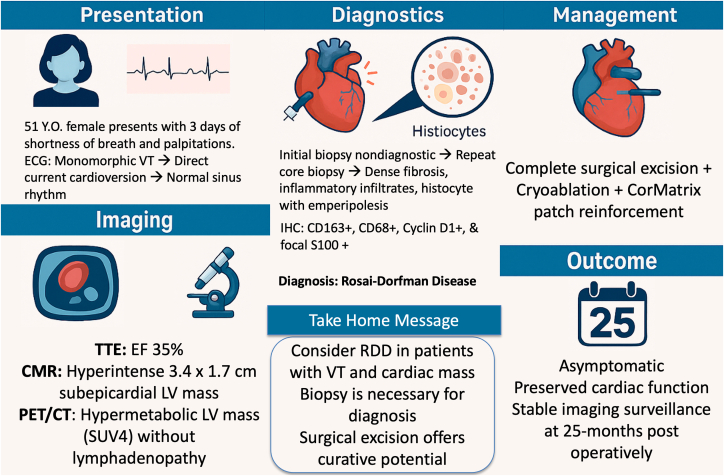
Rare Cardiac Presentation of Rosai-Dorfman Disease: Left Ventricular Mass Causing Ventricular Tachycrdia CMR = cardiac magnetic resonance; ECG = electrocardiogram; EF = ejection fraction; IHC = immunohistochemistry; PET/CT = positron emission tomography/computed tomography; RDD = Rosai-Dorfman disease; SUV4 = standardized uptake value of 4; TTE = transthoracic echocardiography; VT = ventricular tachycardia.

## Funding Support and Author Disclosures

The authors have reported that they have no relationships relevant to the contents of this paper to disclose.
